# MiR-135a inhibits non-small cell lung cancer progression by suppressing RAB1B expression and the RAS pathway

**DOI:** 10.18632/aging.103494

**Published:** 2020-07-25

**Authors:** Ye Tian, Lei Zhang, Qian Yu, Zelong Wang, Xueying Yang

**Affiliations:** 1 Division of Thoracic Surgery, The Fourth Affiliated Hospital of China Medical University, Shenyang 110032, China

**Keywords:** non-small cell lung cancer, progression, miR-135a, RAB1B, RAS pathway

## Abstract

Lung cancer is the most common tumor in China and worldwide. Despite advances in diagnosis and therapy, it still represents the most lethal malignancy in industrialized countries. The study of regulatory noncoding RNAs has deepened our understanding of cancer on the molecular and clinical level. In this article, it showed that miR-135a was aberrantly downregulated in non-small cell lung cancer (NSCLC) cells in comparison with normal bronchial epithelial cells, and the expression of miR-135a inhibited proliferation, invasion and metastasis of NSCLC cells in vitro. Moreover, it was demonstrated that miR-135a inhibited the expression of multiple components (including RAS, Raf1, Rac1 and RhoA) of the RAS pathway via RAB1B, which was a novel target of miR-135a. The expression of miR-135a and RAB1B could effectively predict the clinical outcomes of NSCLC. In summary, miR-135a might function as a suppressor of NSCLC cells, and thus could be used as a potential therapeutic target.

## INTRODUCTION

Lung cancer is the most commonly diagnosed human cancer with a poor prognosis, and it is the leading cause of cancer-related mortality, resulting in approximately 1.77 million deaths worldwide in 2018 [1]. NSCLC accounts for 80% of all lung cancer cases [2]. Standard lung cancer treatments include surgery, chemotherapy, radiation therapy, and targeted therapy. Despite significant improvements in diagnosis and therapy over the last decades, the overall five-year survival rate of lung cancer patients unfortunately remains below 20% because only 16% of patients are diagnosed in the early stage [3]. Therefore, there is an urgent need to identify useful biomarkers and explore novel therapeutic targets.

MicroRNAs (miRNAs) are small and noncoding RNA molecules that regulate gene expression by direct interaction with the 3’-untranslated region (3’-UTR) of corresponding target messenger RNAs (mRNAs) [4]. MiRNAs can regulate a wide range of cellular functions, including proliferation, apoptosis, and differentiation [5]. Recently, it has been revealed that miRNAs dysfunction plays a crucial role in tumorigenesis and cancer progression [6]. Several studies have shown that dysregulated miRNA expression correlates with various human cancers, indicating that miRNAs can act as tumor suppressors or oncogenes [7]. In the previous study, we identified miRNAs including miR-29a/b/c, miR-135a, and miR-203 downregulated in lung cancers, and the analysis results indicated that miR-135a might be involved in a pathway affecting NSCLC [8]. Increasing evidence has shown that miR-135a is involved in some cancers. For instance, miR-135a acts as a suppressor of epithelial ovarian cancer by downregulating homeobox A10 (HOXA10) expression [9]. MiR-135a promotes the apoptosis of classical Hodgkin lymphoma by mediating downregulated expression of Janus kinase 2 (JAK2) and decreasing the anti-apoptotic gene Bcl-xl (BCL2L1) expression [10]. These findings indicated that miR-135a played a vital role in human carcinogenesis. However, the exact roles and mechanisms of miR-135a in lung cancer have not been well elucidated.

In the present study, we demonstrated that miR-135a could inhibit NSCLC cell proliferation and invasion in vitro, and RAB1B was identified as a functional target of miR-135a in NSCLC carcinogenesis. Multiple key players in the RAS pathway, including RAS, Raf1, Rac1, and RhoA, were negatively regulated by miR-135a. Furthermore, miR-135a and RAB1B were proven to have clinical value. Our results promoted the deep understanding of miR-135a as a potential therapeutic target in lung cancer.

## RESULTS

### MiR-135a is downregulated in NSCLC cell lines

**Figure 1:**
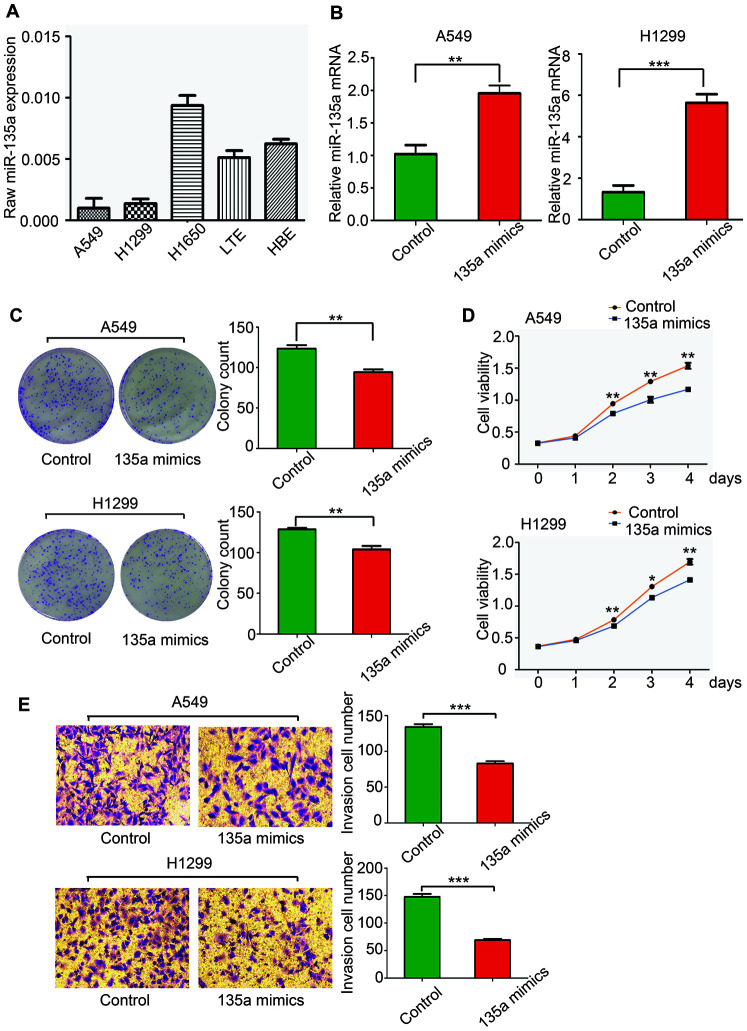
**MiR-135a inhibits human NSCLC cell proliferation and invasion in vitro.** (**A**) Real-time PCR was conducted to quantify the endogenous expression of miR-135a in different NSCLC cells. Assays were performed in triplicate, and expression of U6 was used as a normalization control. (**B**) The effects of miR-135a mimics transfection on A549 and H1299 cells were measured. (**C**) Assessment of the clonogenic potential of miR-135a-transfected A549 and H1299 cells. The colony forming efficiency of miR-135a mimics-transfected cells was less than that of control cells. (**D**) The effects of miR-135a mimics on A549 and H1299 cell proliferation were measured by the CCK-8 assay. (**E**) The invasion assay showed that invasive cells in the miR-135a mimics group were significantly less than those in the control group. Data are expressed as the mean ± sd. * p < 0.05; ** p < 0.01; *** p < 0.001.

We first examined miR-135a expression in a panel of lung cancer cells, which included A549, H1299, H1650, and LTEP-a-2. Compared with that in the immortalized human lung/bronchus epithelial cell line HBE, the expression of miR-135a was lower in A549, H1299, and LTEP-a-2 cell lines, and the difference ranged from a 5/6 decrease in A549 cells to a 1/10 decrease in LTEP-a-2 cells (Figure 1A). This information was consistent with our previous finding that miR-135a expression levels were decreased in lung cancer tissues (8), confirming that miR-135a was decreased in NSCLC cell lines and that it might play an important role in lung cancer progression and metastasis.

### MiR-135a inhibits lung cancer cell proliferation and invasion in vitro

Lung adenocarcinoma cell lines A549 and H1299 were selected due to the low expression of miR-135a for the analysis of effects of miR-135a induction. MiR-135a mimics were used to induce miR-135a expression, and successful overexpression of miR-135a in A549 and H1299 was confirmed by qRT-PCR (Figure 1B). To study the role of miR-135a in tumor cell growth, clone formation and CCK-8 assays were performed. As shown in Figure 1C, there were fewer clones in the miR-135a mimics group than that in the control A549 and H1299 cells. Consistent with these findings, marked suppression of cell viability in the miR-135a mimics group at the 2^nd^, 3^rd^, and 4^th^ day was observed by the CCK-8 assay (Figure 1D).

Cell invasion is a significant factor contributing to cancer progression and metastasis. It involves the migration of tumor cells into contiguous tissues and the degradation of extracellular matrix proteins. We further conducted cell invasion Matrigel assays to measure the directional invasion ability of the tumor cells after ectopic expression of miR-135a in A549 and H1299 cells. The invasiveness of the cells transfected with the miR-135a mimics was dramatically decreased compared with the control cells (Figure 1E). Taken together, our findings supported the idea that the ectopic expression of miR-135a significantly inhibited the proliferation, invasion and metastasis of NSCLC cells in vitro.

### MiR-135a negatively regulates RAB1B and suppresses NSCLC cell growth and invasion

**Figure 2:**
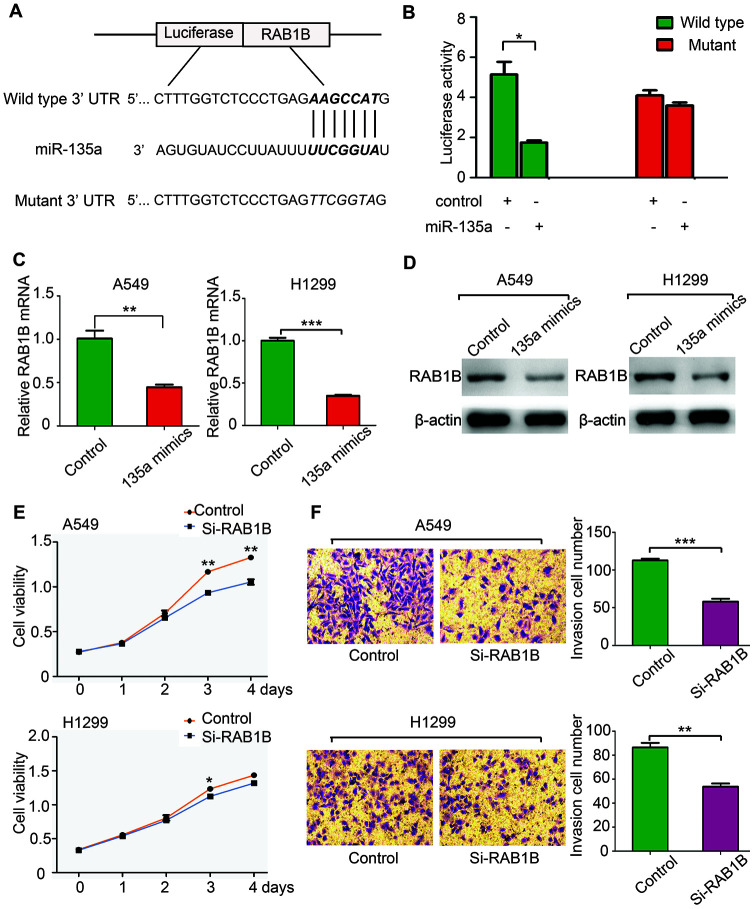
**MiR-135a negatively regulates RAB1B and suppresses NSCLC cell growth and invasion.** (**A**) Putative miR-135a-binding sites in the RAB1B 3’UTR. The nucleotide sequence illustrated the predicted base-pairing between miR-135a and the RAB1B 3’-UTR. (**B**) Luciferase reporter assays demonstrated that the reporter activity of HEK-293T cells decreased by over 50% upon cotransfection of the wild-type RAB1B 3′-UTR reporter construct and miR-135a mimics. (**C**, **D**) Real-time PCR (**C**) and western blotting (**D**) analysis of endogenous RAB1B expression in A549 and H1299 cells after transfection with miR-135a mimic vectors. (**E**, **F**) A549 and H1299 cells were transfected with RAB1B-specific siRNAs and subjected to proliferation (**E**) and invasion (**F**) assays as described above. Data are expressed as the mean ± sd. * p < 0.05; ** p < 0.01; *** p < 0.001.

Computational algorithms TargetScan and PicTar were used for prediction of target genes of miR-135 in this paper, and several candidate genes were identified. Their 3’-UTRs were conjugated with luciferase for reporter assays. RAB1B has a potential effect on cancer progression [11], so we constructed vectors containing the wild-type or mutant 3’-UTR of RAB1B fused directly with the downstream of the luciferase gene (Figure 2A). The wild-type or mutant vector was transfected into HEK-293T cells with miR-135a expressed or vector controlled. As shown in Figure 2B, miR-135a significantly reduced the luciferase activity of wild-type RAB1B 3’-UTR (more than 50%), yet had little effect on the luciferase activity of mutant RAB1B, suggesting that miR-135a could directly or indirectly bind to the 3’-UTR of RAB1B. Then real-time PCR and western blotting were performed to verified that RAB1B expression could be suppressed by miR-135a. As expected, it was found that overexpression of miR-135a in both A549 and H1299 cells reduced the mRNA level of RAB1B by more than 50% (Figure 2C). Western blotting analysis indicated that forced expression of miR-135a significantly inhibited endogenous RAB1B protein expression (Figure 2D). The above results demonstrated that miR-135a negatively regulates the expression of RAB1B through a seed sequence interaction.

To explore the biological function of RAB1B in lung cancer cells, two specific siRNAs against RAB1B mRNA were transduced into A549 and H1299 cells. Cell proliferation assays showed that inhibition of RAB1B significantly suppressed A549 and H1299 cell growth (Figure 2E). Moreover, a Transwell assay with Matrigel coating revealed that si-RAB1B inhibited A549 and H1299 cell invasion (Figure 2F). These results suggested that miR-135a negatively regulated RAB1B and suppressed the growth and invasion of NSCLC cells.

### MiR-135a inhibits the expression of multiple components of the RAS pathway via RAB1B

**Figure 3:**
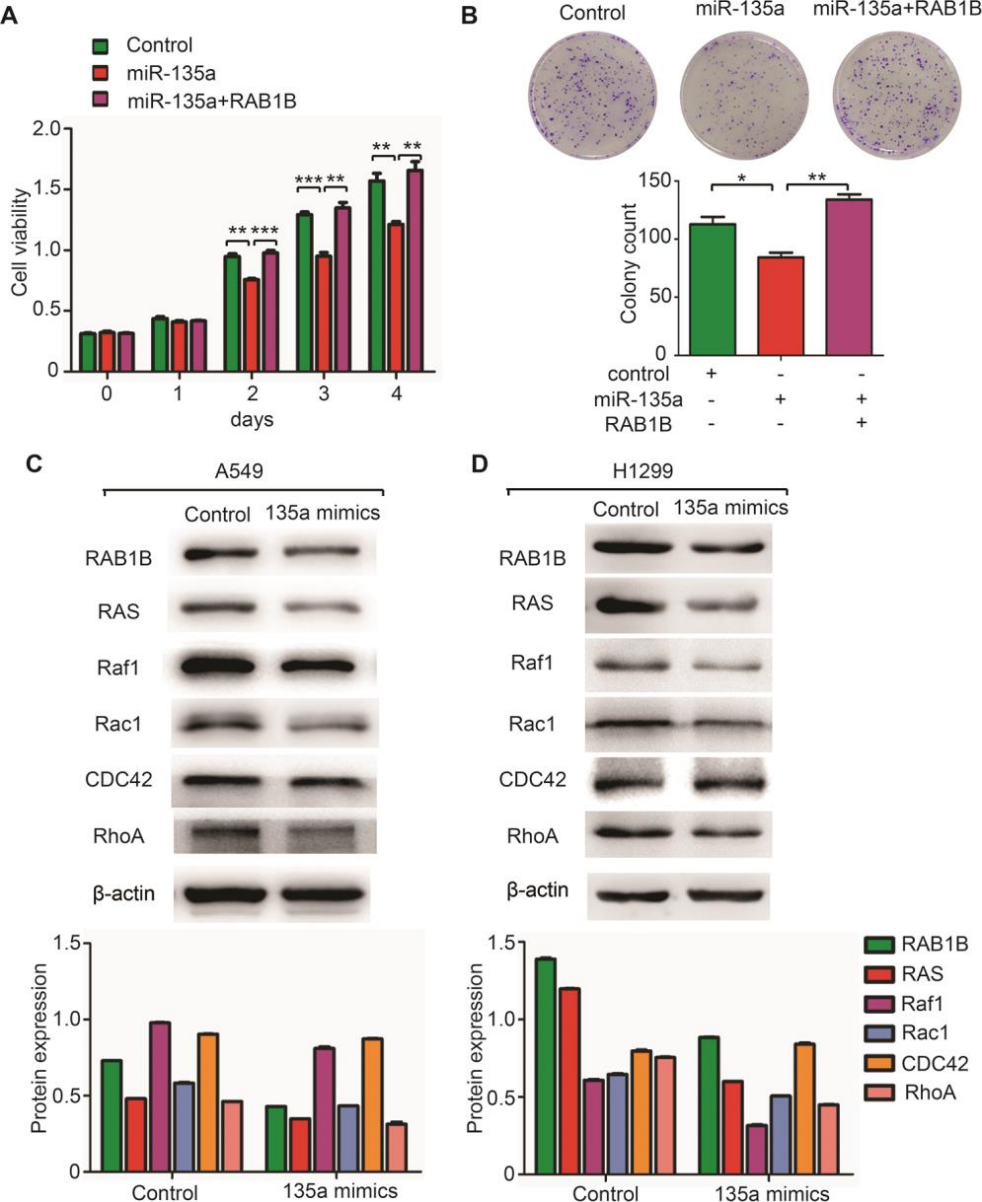
**MiR-135a inhibits the expression of multiple components of the RAS pathway.** (**A**, **B**) Overexpression of RAB1B antagonized the effects of miR-135a mimics on (**A**) cell viability and (**B**) colony forming ability. (**C**, **D**) Western blotting analysis of the RAB1B and RAS pathway components in (**C**) A549 and (**D**) H1299 cells transduced with miR-135a mimics. Due to the overall contrast adjustment, some parts are no background in figure 3C and 3D.

If RAB1B is indeed a functional target of miR-135a, the introduction of RAB1B into miR-135a-expressing cells should antagonize the effects of miR-135a. To test this hypothesis, we cotransfected A549 cells with miR-135a mimics and a RAB1B expression vector. Interestingly, a cell proliferation assay demonstrated that overexpression of RAB1B enhanced the proliferation of miR-135a-expressing cells (Figure 3A). Furthermore, the inhibition of miR-135a on colony formation was antagonized by enforced expression of RAB1B (Figure 3B). However, RAB1B reintroduction did not rescue the invasion suppression induced by miR-135a. These findings indicated that RAB1B was a functional mediator of miR-135a in NSCLC cells.

As a member of the RAB protein family, RAB1B is a low molecular mass monomeric GTPase existing in cytoplasm, and facilitates intracellular vesicular trafficking in the endocytic pathway by regulating the RAS pathway, which plays an important role in tumorigenesis in mammals [12]. The central axis of the RAS pathway is a kinase cascade that includes RAS as well as the downstream factors Raf1, Rac1, CDC42 and RhoA. The results of this study confirmed the regulation of the RAS pathway by miR-135a in A549 and H1299 cells. Overexpression of miR-135a reduced the expression levels of RAB1B protein as well as downstream RAS, Raf1, Rac1 and RhoA proteins, but it failed to alter CDC42 protein expression (Figure 3C, 3D). These findings indicated that miR-135a inhibited the expression of multiple targets in the RAS pathway via RAB1B.

### Clinical correlation of miR-135a and RAB1B in NSCLC

**Figure 4:**
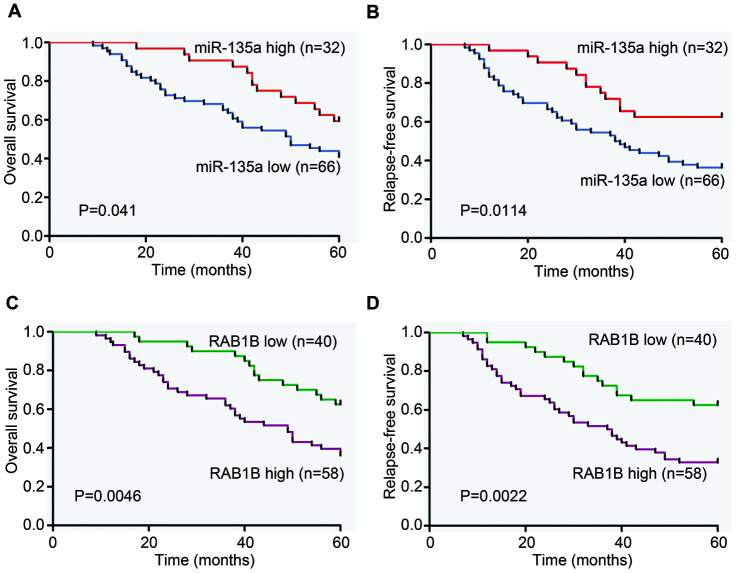
**Clinical analysis of the expression of miR-135a and RAB1B in NSCLC patients.** Expression levels of miR-135a and RAB1B were examined in serial dissections of primary tumor and para-carcinoma specimens from 98 NSCLC patients who underwent surgical resections. (**A**, **B**) Kaplan-Meier plots of (**A**) overall survival and (**B**) relapse-free survival in high- and low-risk groups based on miR-135a expression levels. (**C**, **D**) Kaplan-Meier estimates of (**C**) overall survival and (**D**) relapse-free survival according to RAB1B expression levels. P-values were obtained from log-rank tests.

To further delineate the clinical significance of downregulated miR-135a expression in lung cancer progression, we examined the relationship of the miR-135a expression profile with overall survival and relapse-free survival in tumor specimens from 98 lung cancer patients. MiR-135a expression was measured by real-time RT-PCR, and Kaplan-Meier and Log rank test analysis showed that low levels of miR-135a expression were closely associated with decreased overall survival (P=0.041) and relapse-free survival (P=0.0114) (Figure 4A, 4B). Cox proportional hazard regression analysis demonstrated that the overall survival (HR=0.538) and relapse-free survival (HR=0.457) of this cohort were correlated with miR-135a expression levels. Meanwhile, the relationship between the expression of the miR-135a and the downstream target gene RAB1B was also investigated. Kaplan-Meier and Log rank test analysis showed that high expression of RAB1B was associated with poor overall survival (P=0.0046) and relapse-free survival (P=0.0022) (Figure 4C, 4D). Cox proportional hazard regression analysis indicated that RAB1B was a risk factor (OS: HR=2.246, 95% CI = 1.231 to 4.097, P=0.008; RFS: HR=2.531, 95% CI=1.373 to 4.667, P=0.003), while miR-135a was considered as a protective factor, suggesting that patients with a high expression level of RAB1B may have a lower mortality. The above results indicated that the survival of NSCLC patients was strongly related to miR-135a and its target RAB1B.

## DISCUSSION

**Figure 5:**
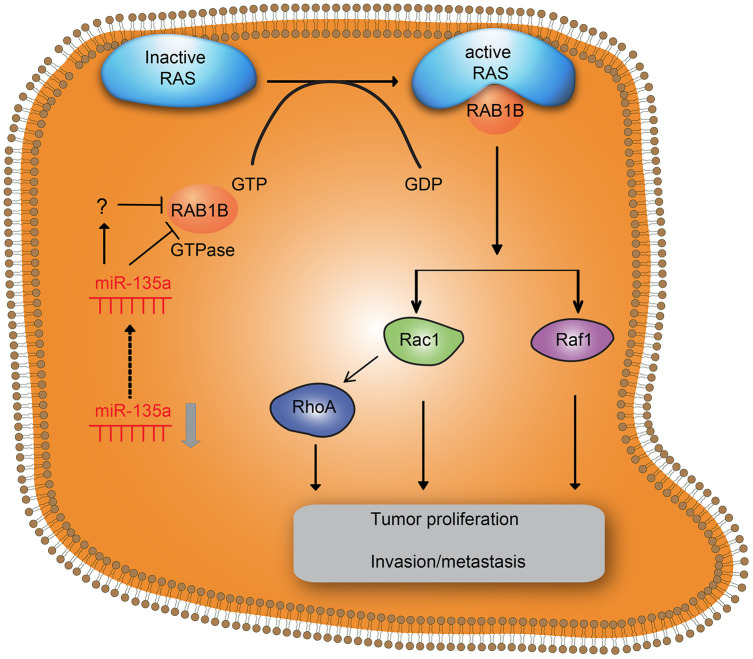
**Effects of downregulation of miR-135a on RAB1B and the RAS signaling pathway in NSCLC cell progression.** Downregulation of miR-135a increased the expression of downstream target gene RAB1B and RAS pathway members, thereby promoting proliferation and invasion/metastasis of NSCLC.

The poor survival and prognosis of NSCLC partly result from relapse, metastasis and resistance to chemotherapy, radiotherapy and target therapy [2]. Therefore, clarification of the molecular pathogenesis of NSCLC is crucial for developing effective intervention strategies for this disease. The ability of a single miRNA sequence to regulate numerous target genes enhances the phenotypic effects of the miRNA [13]. Several miRNAs are known to promote tumorigenesis and progression by regulating distinct targets, as is the case for miR-200 and miR-34a in the development of breast cancer and meningioma [14, 15]. Our study provided strong evidence that miR-135a was expressed at low levels in lung cancer cells and tissues, and it inhibited lung cancer cell growth, invasion and metastasis by suppressing the expression of RAB1B and multiple key components of the RAS pathway (Figure 5).

MiRNAs are endogenous RNA molecules that mainly function in posttranscriptional gene expression by binding to the 3’-UTR of target genes, and have been identified as critical regulators in cancer initiation and metastasis [16]. Previous evidence has indicated that miR-135a is downregulated in malignant glioma [17] and ovarian cancer [9]. In addition, Hodgkin lymphoma patients with low miR-135a expression often have a higher probability of relapse and shorter disease-free survival [10]. In contrast, miR-135a has been reported to promote cervical cancer [18] and hepatocellular carcinoma cell migration and invasion [19]. These observations suggest that the role of miR-135a is dependent on the origin of tumor cells. Our study noticed the greatly decreased expression of miR-135a in NSCLC cell lines and tumor tissues, which led to poor overall survival and relapse-free survival of NSCLC patients and might served as a clinical prognostic factor. These data implied that miR-135a played a key role in regulating several signaling pathways, and thereby could be potentially used for the development of lung cancer therapy.

A series of miR-135a target genes involved in various signaling pathways were identified by predicted gene analysis in this paper. It is worth noting that increased expression levels of miR-135a led to a decrease in RAB1B expression and thus lower proliferation and invasion ability of the tumor cells. RAB1 is a small GTPase confirmed to be able to mediate vesicular trafficking between the endoplasmic reticulum (ER) and Golgi apparatus [20]. In addition to promoting vesicular trafficking, the RAB1 protein also has the functions of nutrient sensing and signaling [21], cell migration [22] and autophagy regulation, as shown by a growing body of evidence [23]. It has been reported that reduced expression of RAB1B is correlated with poor clinical outcomes in breast cancer [11] and colon cancer [24]. However, in this study, RAB1B was shown to facilitate the proliferation and invasion of lung cancer cells, and high RAB1B expression in tumor tissues was related to poor overall survival and relapse-free survival of NSCLC patients. Furthermore, RAB1A, a homolog of RAB1B, was proven to be upregulated in lung cancer and associated with T stage [25]. Generally, miR-135a might inhibit cancer proliferation, invasion and metastasis by downregulating RAB1B in lung cancer cells.

Interestingly, other researchers have established that RAB proteins interact with RAS, a small guanosine triphosphate binding protein, which plays an important role in signal transduction pathways that influence cellular proliferation, apoptosis, and cytoskeletal organization (12). A further analysis of a series of miR-135a target genes that belong to the RAS signaling pathway found that miR-135 might suppress tumor by regulating the RAS-MAPK pathway [26]. RAS pathway constitutive activation is common in some human cancers. It is mainly composed of a kinase cascade that includes RAS, Raf1, Rac1, CDC42, and RhoA. Our results showed that miR-135a suppressed the RAS pathway by downregulating RAS, Raf1, Rac1, and RhoA protein expression. Therefore, miR-135a might be an effective way to control RAB1B expression and block the RAS pathway.

Finally, this study has a number of limitations, which caution us about the extrapolation of our findings. For instance, miR-135a has been reported to inhibit tumor metastasis and angiogenesis by targeting the FAK pathway in gastric cancer [27]. The effects of miR-135a on the JAK2/STAT5 pathway lead to apoptosis and survival of classical Hodgkin lymphoma [10]. This evidence indicates that miR-135a is involved in the complicated crosstalk in tumor cells. Thus, it should be evaluated in a more complex genetic background. Another limitation of our study is the absence of experiments testing miRNA effects in a specific dose-dependent manner to make accurate observations. In addition, RAB1B reintroduction rescued the proliferation and colony formation, but failed to impact the invasion of NSCLC cells, which was possibly attributed to the regulatory effect of miR-135a on some genes other than RAB1B. RAB1B might be not the pivot point of this invasive pathway, which explained why RAB1B reintroduction did not rescue the invasion suppression induced by miR-135a. Thus, such experiments are required to be performed in future studies.

In conclusion, miR-135a is significantly downregulated and closely related to NSCLC progression. Functional and mechanistic studies indicate that activated miR-135a can inhibit cell growth and invasion by suppressing RAB1B expression and the RAS pathway. These findings facilitate a better understanding of the molecular pathogenesis of NSCLC and suggest that miR-135a may be a candidate for cancer treatment.

## MATERIALS AND METHODS

### Ethics statement

All patients agreed to participate in the study and signed an informed consent form. Both the study and consent form were approved by the Ethics Committee of the Fourth Affiliated Hospital of China Medical University and complied with the Declaration of Helsinki.

### Cell lines, culture, and tissue sample

The human lung cancer cell lines (A549, H1299, H1650, and LTEP-a-2), normal human bronchial epithelium cell HBE and HEK-293T cells were obtained from the Cell Bank of the Chinese Academy of Science (Shanghai, China). Cells were cultured in Dulbecco’s modified Eagle’s medium supplemented with 10% fetal bovine serum (HyClone, Logan, Utah, USA), 100 IU/ml penicillin and 100 μg/ml streptomycin in a humidified atmosphere at 37°C with 5% CO_2_. Cells were grown on sterilized glass Petri dishes and detached for subculture using 0.25% trypsin (Gibco, Carlsbad, CA, USA). Human miR-135a mimics was synthesized by RiboBio (Guangzhou, China). Plasmid vectors expressing RAB1B and si-RAB1B were constructed by GeneChem (Shanghai, China). Lipofectamine 2000 reagent (Invitrogen, Carlsbad, USA) was used to transfect cells according to the recommendations of the manufacturer.

Frozen lung cancer specimens from 98 patients who underwent surgical resection of NSCLC were collected from the Fourth Affiliated Hospital of China Medical University. None of the patients had received adjuvant chemotherapy. Fresh tissue samples were stored at -80°C for qRT-PCR.

### TaqMan RT-PCRs for miRNA expression

Total RNA was extracted with TRIzol reagent (Invitrogen, CA). Complementary DNA synthesis was performed using the PrimeScript RT reagent Kit (Takara, Dalian, China) according to the manufacturer’s instructions. The levels of RAB1B transcript were measured by forward primer GGACTTCAAGATCCGAACCAT and reverse primer ATACACCACGATGATGCCA. β-Actin was used as an internal control and amplified with forward primer AGACCTGTACGCCAACACAG and reverse primer CGGACTCGTCATACTCCTGC. The miRNA primer specific for miR-135a was designed by RiboBio (Guangzhou, China). U6 small nuclear RNA was used for normalization.

### Western blotting

Western blot analysis was carried out using standard methods. Total protein was extracted from the cultured cells or tumor tissues with lysis buffer. Protein concentration was determined by using a BCA method. The protein sample was then separated by 8-10% SDS-PAGE and transferred onto PVDF membranes. The membranes were incubated with primary antibodies overnight at 4°C. Membranes were probed with anti-RAB1B (1:1000), anti-Ras (1:5000), anti-Raf1 (1:1000), anti-Rac1 (1:500), anti-RhoA (1:5000), anti-CDC42 (1:1000), anti-β-actin (1:5000), and HRP conjugated anti-mouse (1:5000) and anti-rabbit (1:5000) (Abcam, Burlingame, CA).

### Cell proliferation and colony formation

Cell proliferation was determined by a Cell Counting Kit-8 (Dojindo, Shanghai, China) according to the manufacturer's instructions. For colony formation assays, 500 cells were plated into 6-cm cell culture dishes and incubated for 14 days. Cells were then stained with violet (Sigma-Aldrich, Shanghai, China), and the numbers of colonies per well were counted.

### In vitro invasion assay

Transwell chambers were coated with BD Matrigel. Next, 5×10^4^ cells were seeded into the upper chamber with serum-free medium. After 48 h of incubation at 37°C, the cells on the top surface of the chambers were completely removed by cotton swabs, and the cells on the bottom surface that had crossed the Matrigel were fixed with 4% paraformaldehyde for 15 minutes and stained with crystal violet. To calculate tumor cell invasion, cells adhering to the lower membrane were counted using an inverted microscope (Olympus, Tokyo, Japan).

### Dual luciferase assays

The wild-type 3′-UTR of RAB1B mRNA containing predicted miR-135a binding sites and the mutant 3-′UTR of RAB1B were inserted into the psi-CHECK-2 vector. HEK-293T cells were seeded in 96-well plates and cotransfected with wild-type or mutant RAB1B 3′-UTR luciferase reporter and miR-135a mimics or control using Lipofectamine 2000. Cells were harvested 48 h posttransfection and then assayed with the Dual Luciferase Assay System (Promega, WI, USA) according to the manufacturer’s instructions. Firefly luciferase values were normalized to the Renilla signal, and the firefly/Renilla ratios were reported.

### Statistical analysis

For cell line models, data are presented as the mean ± sd. The differences between two groups were assessed using Student’s t-test, and the Kaplan–Meier method was used to estimate survival. Differences in survival between two groups were analyzed using the log-rank test. Multivariate Cox proportional hazard regression analysis with stepwise selection was used to evaluate independent prognostic factors associated with patient survival. All statistical calculations were performed using SPSS for Windows software. A p value less than 0.05 was considered statistically significant.
